# An Introduction to B-Cell Epitope Mapping and In Silico Epitope Prediction

**DOI:** 10.1155/2016/6760830

**Published:** 2016-12-29

**Authors:** Lenka Potocnakova, Mangesh Bhide, Lucia Borszekova Pulzova

**Affiliations:** ^1^Laboratory of Biomedical Microbiology and Immunology, Department of Microbiology and Immunology, The University of Veterinary Medicine and Pharmacy in Kosice, 041 81 Kosice, Slovakia; ^2^Institute of Neuroimmunology of Slovak Academy of Sciences, 845 10 Bratislava, Slovakia

## Abstract

Identification of B-cell epitopes is a fundamental step for development of epitope-based vaccines, therapeutic antibodies, and diagnostic tools. Epitope-based antibodies are currently the most promising class of biopharmaceuticals. In the last decade, in-depth in silico analysis and categorization of the experimentally identified epitopes stimulated development of algorithms for epitope prediction. Recently, various in silico tools are employed in attempts to predict B-cell epitopes based on sequence and/or structural data. The main objective of epitope identification is to replace an antigen in the immunization, antibody production, and serodiagnosis. The accurate identification of B-cell epitopes still presents major challenges for immunologists. Advances in B-cell epitope mapping and computational prediction have yielded molecular insights into the process of biorecognition and formation of antigen-antibody complex, which may help to localize B-cell epitopes more precisely. In this paper, we have comprehensively reviewed state-of-the-art experimental methods for B-cell epitope identification, existing databases for epitopes, and novel in silico resources and prediction tools available online. We have also elaborated new trends in the antibody-based epitope prediction. The aim of this review is to assist researchers in identification of B-cell epitopes.

## 1. Introduction

Antigen-antibody interaction is a key event in humoral immune response to invading pathogen. A specific antibody (Ab) recognizes antigen (Ag) at discrete regions known as antigenic determinants or B-cell epitopes. B-cell epitopes can be defined as a surface accessible clusters of amino acids, which are recognized by secreted antibodies or B-cell receptors and are able to elicit cellular or humoral immune response [1].

Most of the Ag surface may become part of epitopes after recognition with antibodies and the exact selection mechanism why certain antigen regions become B-cell epitopes is not fully understood [2]. The classification of antigenic determinants into epitopes and nonepitopes ignoring the antigen reconfiguration in Ag-Ab complex may not accurately reflect biological reality [3]. The accurate identification of B-cell epitopes constitutes a basis for development of antibody therapeutics [4], peptide-based vaccines [4, 5], and immunodiagnostic tool [6].

Based on the spatial structure B-cell epitopes can be categorized as a continuous (linear or sequential) and discontinuous (nonlinear or conformational) epitopes; in the latter case amino acid residues are in close contact due to the three-dimensional conformation [7]. The minimal amino acid sequence (contact residue span) required for proper folding of the discontinuous epitope in native proteins may range from 20 to 400 amino acids. It is generally believed that most of identified linear antigenic determinants are parts of the conformational B-cell epitopes [8–10]. Using a less stringent definition for continuity, it was found that the majority of discontinuous epitopes (over 70%) are composed of 1–5 linear segments of lengths of 1–6 amino acids [10].

The experimental methods developed to identify the epitopes can roughly be divided into structural and functional studies. The X-ray crystallography can exactly locate the position of epitope within the protein structure but is laborious, time consuming, costly, technically difficult, and not applicable for all antigens [11]. Some of the commonly used methods for functional B-cell epitope mapping are screening of antigen-derived proteolytic fragments or peptides for antibody binding and testing the Ag-Ab reactivity of mutants (site-directed or randomly mutated) [11]. Other techniques like display technologies and mimotope analysis have also become acceptable alternative choices for epitope mapping thanks to their relative cheapness, flexibility, and speed [12, 13].

Rubinstein and colleagues proposed a null hypothesis that the surface of the antigen is homogeneously antigenic. With the large-scale statistical analysis of Ag-Ab cocrystals derived from the protein databases, they were able to define physicochemical, structural, and geometrical aspects of epitopes and concluded that epitopes are clearly distinguishable from the remaining antigen surface [10]. In another study, Kringelum and coworkers described B-cell epitope as a flat, elongated, oval shaped bundle with unorganized secondary structure [14]. Thanks to the comprehensive experimental studies and in silico analyses conducted hitherto, it is possible to defined the features distinguishing epitope from nonepitope. The majority of epitopes span 15–25 residues and an area of 600–1000 Å^2^ organized in loops. The epitope surface accessibility is common feature. Sequence of the epitopes is enriched with Y, W, charged, and polar amino acids (amino acids with exposed side chains) and with specific amino acid pairs. The Ag-Ab interaction occurs without preference for a specific CDR loop and involves epitope compression [10]. In recent years, it was shown that the differences between residues within epitopes and other residues are not substantial and amino acid composition is not sufficient for differentiating between epitopes and nonepitopes (reviewed in [2]).

Advancement in the epitope mapping technologies hand in hand with bioinformatics has greatly contributed to developing immunoinformatics, which involves application of computational methods in immunology to unveil structures of antibody, B-cell, T-cell, and allergen, prediction of MHC binding, modelling of epitopes, and analysis of immune networks. Several algorithms have been developed to predict B-cell epitopes from their sequence or structure [15–18]. The early prediction methods were focused on the identification of linear epitopes through propensity scale. To improve prediction performance, methods based on machine learning such as Hidden Markov Model [19], recurrent neural network [20], and support vector machine [21] were developed. Despite this advancements, there are still a limited number of methods that predict discontinuous epitopes, and they need combination of the information, for example, amino acid statistics, spatial information, and surface exposure [22].

Identification B-cell epitopes is extensively employed in the development of diagnostic tests, therapeutics and vaccines [23–26]. Use of epitope mapping in the drug development is reviewed earlier [27]. In spite of advances in B-cell epitope mapping, it is important to note that antibodies raised against peptides often lack the ability to bind native proteins due to unstructured nature of the peptide [28].

The main purpose of this review is to provide researcher with the general knowledge about existing methods of B-cell epitope mapping and short overview of epitope databases, recently used prediction methods, and publicly available tools.

## 2. B-Cell Epitope Mapping

Most of the existing methods for epitope mapping (structural and functional approach) are expensive, laborious, time consuming, and often fail to identify all epitopes. Structural epitope mapping methods interpret the protein structure comprising residues in direct contact with an antibody but often fail to reveal contribution of amino acids in binding strength. The identification and characterization of residues important for binding within structurally defined antigenic determinant are the aim of functional epitope mapping tools.

The most accurate method for structural epitope mapping is X-ray crystallography of Ag-Ab complexes and is often regarded as the only method to define a structural epitope [29]. Among “wet” lab methods this technique is a guarantee of precise identification of both continuous and discontinuous epitopes and provide information about strength of binding [30–32]. Bacterial or viral antigens, especially small soluble proteins, are ideal for crystallography. However, the X-ray crystallography is limited by quality of cocrystals and electron density of the antibody [33]. Recently developed freely available program FTProd can be used as computational alternative to expensive and time consuming X-ray crystallography [34]. Nuclear magnetic resonance (NMR) has also potential to replace traditional X-ray crystallography. This approach provides data about the structure, dynamics, and binding energy of Ag-Ab complex and is performed in solution where no crystals are needed. However, NMR is limited to small proteins and peptides (<25 kDa) [35]. Saturation transfer difference NMR (reviewed in [36]) and antibody inhibition of hydrogen-deuterium exchange in the antigen are other two methods capable of mapping epitope regions with moderate resolution [37]. The electron microscopy (EM) can also be used for epitope localization; however it is a low-resolution structural method that is utilized on larger antigens (e.g., whole viral particles) [38]. Unfortunately, this method is unable to detect contact residues and can be used for confirmation of surface accessibility of the epitope [39]. An alternative, cryoelectron microscopy allows observations of rapidly frozen Ag-Ab complexes in physiological buffers avoiding the need for stains and fixatives [40, 41].

The methods for functional epitope mapping can be divided into four main groups: competition methods, antigen fragmentation methods, modification methods, and methods using synthetic peptides or peptide libraries [42]. Competition methods have low-resolution degree of mapping and are commonly used to determine whether two different monoclonal antibodies (mAbs) can bind to antigen at the same time or whether they compete with each other for the same epitope [11]. Most of the functional methods are based on the ability to detect binding of antibody to antigen fragments, synthetic peptides, or recombinant antigens (including mutated variants, antigens arrayed by in situ cell-free translation, and/or expressed using selectable systems such as phage display). In the binding assays, peptides are immobilized on solid support and binding of antibody is detected by western blot, dot blot, and/or ELISA. This approach does not require expensive equipment and is able to quantify the immune response towards a specific epitope. The dot blot requires the purified molecule to be spotted on the membrane and is mainly used for qualitative detection [43]. The peptides can be synthesized on pins (PEPSCAN®), on a cellulose membrane support (SPOT method®), or on peptide microarrays [44, 45]. Such techniques simplify the handling of large numbers of peptides and eliminate the need for identification of positive peptides by sequencing or by mass spectrometry. Binding assays were successfully used in identification of epitopes in several viruses, bacteria, fungi, parasites, and human diseases (reviewed in [27]).

### 2.1. Mutagenesis

Mutagenesis is a rapid epitope mapping method that relies on the fact that substitution of individual residue/s (hot-spot/s) that constitutes a functional epitope causes loss of antibody binding. Hot-spots (most frequently Tyr, Arg, and Trp) are energetically important residues and comprise only a fraction of the complete protein-protein interface area [39]. The protein library can be generated by either random or site-directed mutations. The combination of mutagenesis approach with display techniques enables screening of many hundreds or thousands of mutated proteins (reviewed in [46]). The saturation mutagenesis, another versatile tool, replaces amino acid residue at specific position with all 20 naturally occurring residues. However, in some cases the loss of immunoreactivity due to the disruption of antigenic structure complicates the interpretation of the results.

The majority of epitope contacts in Ag-Ab complex occur through amino acid side chains [10]. Alanine scanning mutagenesis provides a controlled method to define the contributions of each residue's side chain to Ab binding by alanine sequential substitution (causing truncation of side chains to *β*-carbon without additional flexibility of protein backbone) for each nonalanine residue one at a time. Although this mapping strategy may not identify every residue in contact with an antibody, the critical residues identified using this approach represent amino acids whose side chains make the highest energetic contributions to the paratope-epitope interaction [47]. The generation of combinatorial libraries of displayed alanine mutations significantly accelerates the functional mapping of epitopes. Computational alanine scanning can also rapidly calculate the effect of alanine mutation on a binding free energy in protein-protein complex using a simple free energy function (available at http://robetta.bakerlab.org/alaninescan) [48].

The combinatorial mutagenesis enables identifying residues, which are not critical for binding but contribute to the formation of epitope or establish multiple individually weak interactions with paratope. This strategy is based on combinatorial randomization of a discrete antigenic region and grouping of mutated residues (primary sequence proximity) to maximize the chances of underscoring combined effects mediated by neighboring residues [49].

Another technique in mutagenesis, a shotgun mutagenesis, enables identification of both linear and conformational epitopes with mapping rates of over 20 epitopes/month. This high-throughput strategy is based on large-scale mutagenesis, where each clone bears a defined amino acid mutation (such as an alanine substitution) and direct cellular testing for mAb reactivity of natively folded proteins (proper oligomerization, disulphide bonds, glycosylation, and other posttranslational modifications). Shotgun mutagenesis has been used to map over 250 mAbs targeting dengue, chikungunya, and hepatitis C viruses, with additional mAb epitopes mapped on hepatitis B virus, respiratory syncytial virus, and HIV (reviewed in [50]).

### 2.2. Display Techniques

Display technologies, best exemplified by phage and yeast display, provide a powerful technique for epitope mapping. Display techniques have become acceptable alternative for epitope mapping due to their relative cheapness and quickness [12]. The principle of display methods is based on testing the binding capacity of a variety of peptides displayed on the display platforms (tethering of proteins to ribosomes-mRNA complex, or to the surface of phage, bacteria, mammalian, insect, or yeast cells) to the monoclonal antibody of interest through the affinity selection method of biopanning.

One of the most frequent and popular display methods for epitope mapping is phage display. Construction of phage display peptide libraries (displaying >10^9^ of peptides) represents popular way of generation of antigenic fragments which are screened for antibody binding [12]. This powerful approach involves fusion of the foreign DNA fragments with the filamentous phage gene coding coat protein (e.g., pIII, pVI, pVII, pVIII, and pIX). The bacteriophage M13 or lytic alternatives such as T4, T7, and P4 bacteriophages or lambda phage are usually used as model viruses for phage display. Random peptide phage libraries (combinatorial libraries) and gene or genome fragment phage libraries are commonly used techniques for epitope identification (reviewed in [29]).

## 3. B-Cell Epitope Databases

Thanks to the technological advances in genomics, proteomics, and epitope mapping techniques, huge amounts of data are being generated and are necessary to organize in a searchable form. B-cell epitope databases provide a training set for evaluation of existing epitope prediction methods and constitute platform for development of novel and better algorithms for prediction. The B-cell epitope databases can be classified as multifaceted database such as IEDB and AntiJen, B-cell oriented database such as BciPep, Epitome, and SDAP, and single pathogenic organism oriented database such as the HIV Molecular Immunology Database, FLAVIdB, and Influenza Sequence and Epitope Database. It has to be mentioned that most of the available databases include peptide/s recognized by the receptors of the adaptive immune system and/or amino acid residues of antigen that are in close contact with antibody (structural epitopes) and lack important epitope information such as a detailed molecular characterization of epitopes and the mention of contact residues that make energetic contributions to binding. The databases that collect B-cell epitope are listed in Table 1.

The Immune Epitope Database (IEDB) is a comprehensive resource aimed to catalogue experimentally determined B-cell and T-cell epitopes from human, nonhuman primates, and other animal species along with the experimental contexts. It captures epitopes related to category A-C pathogens, emerging and reemerging pathogens, allergens, and autoantigens. IEDB contains epitopes derived from the peer-reviewed literature, patent applications, direct submission, and other publicly available databases, for example, FIMM [51], HLA Ligand database [52], and MHC binding database [53]. IEDB also provides tools for the prediction of linear B-cell epitopes from protein sequence including amino acid scales and HMMs, DiscoTope, ElliPro, Paratome, and PIGS. The database houses epitope conservancy analysis tool for determination of the degree of epitope conservation or variability, tool for analysis of population coverage, or tool for localization of epitope in 3D structure of antigen. The IEDB-3D catalogues T-cell and B-cell epitopes and MHC ligands with accompanied functional assays and immunologically relevant information derived from PDB and provides calculation of intermolecular contacts and interface areas [54]. An application, EpitopeViewer, allows visualization of the antigen structures and is fully embedded in IEDB-3D [55].

AntiJen v2.0 (developed from JenPep) contains quantitative binding data for peptides binding to MHC ligand, TCR-MHC complexes, T-cell epitopes, TAP (a transporter associated with the MHC class I restricted antigen processing), and B-cell epitopes. It also contains immunological protein-protein interactions and biophysical data such as diffusion coefficient and cellular data [56, 57]. AntiJen is linked to protein database Swiss-Prot, NCBI, MPID, PDB, and PubMed, which enables further in-depth cross referencing. The aim of AntiJen is to integrate quantitative kinetic, thermodynamic, and biophysical data, with functional and cellular information, which can be used in immunology and immunovaccinology [58]. AntiJen does not allow downloading of the data.

Conformational Epitope Database (CED) provides a manually curated dataset of conformational epitopes that can be used to evaluate existing epitope prediction methods and develop new and better algorithms for prediction [59]. This database has limited size and contains only high quality clearly defined conformational epitopes collected from published peer-reviewed articles. The database implies additional information, such as residues dispatching, localization, immunological properties, source antigen, and corresponding antibody of the epitope. CED is hyperlinked to other databases (e.g., Swiss-Prot, PDB, KEGG, or PubMed). Conformational epitopes with corresponding PDB structures can be viewed interactively in the context of the Ag-Ab complex, antigen structure, or known theoretical model that can help to identify important structural features. The semiautomatic database Epitome collects structure-inferred antigenic residues in proteins that are involved in interaction with residues on antibody CDRs, and it provides information of corresponding paratope [60]. It serves for detailed description of residues with and enables visualization of three-dimensional structure of Ag-Ab complex derived from PDB through Jmol tool [60].

The comprehensive database BciPep provides dataset of experimentally validated linear B-cell epitopes derived from literature and other publicly available databases [61].

In BciPep, B-cell epitopes are categorized into three classes: immunodominant (2-3-fold enhancement of anti-peptide antibody synthesis compared to reference protein or control, e.g., BSA or KLH), immunogenic (onefold enhancement of anti-peptide antibody synthesis compared to reference protein or control, e.g., BSA or KLH), and null-immunogenic (no difference observed when compared to reference protein or control, e.g., BSA or KLH). The database provides information (isotype and name/number) about anti-peptide antibodies produced against an epitope and their neutralization potential. The database is linked with Swiss-Prot, PDB, MHCBN, and PubMed [61].

Structural Epitope Database (SEDB) contains 3D complexes of B-cell, T-cell, and MHC bound molecules and shows Ag-Ab interaction plot. SEDB collects related information of epitopes, like gene-ontology information, Ag-Ab interaction graph, and epitopes location in protein with interaction data, which are missing in currently available epitope databases [62].

Structural Database of Allergenic Proteins (SDAP) contains sequences, structures, and IgE epitopes of allergenic proteins and offers additional computational tools for structural studies. SDAP enables allergen-peptide matching for the detection of novel allergens and the cross-reactivity between known allergens [63].

Databases oriented on single pathogenic organism have been developed to target vaccine design. The HIV Molecular Immunology Database collects cytotoxic, helper T-cell epitopes and B-cell epitopes in annotated and searchable form and offers several generic data analysis tools. FLAVIdB is a comprehensive database of antigens from* Flavivirus* spp. derived from external databases (GenPept, UniProt, IEDB, and PDB) and corresponding literature. It contains flavivirus antigen sequences, T-cell epitopes, B-cell epitopes, and molecular structures of the dengue virus envelope protein. Database is equipped with tools for block entropy analysis and flavivirus species classification [64].

## 4. In Silico B-Cell Epitope Prediction

Correlation between B-cell epitope localization and physicochemical properties (e.g., hydrophilicity, solvent accessibility, flexibility, turns, polarity, antigenicity, and surface exposure), has been demonstrated in several studies (reviewed in [65]). Earlier prediction methods were monoparametric (based on single residue property or propensity scale) calculating average propensity value along a sliding window [66–68]. It was demonstrated that methods based on propensity profiling yield poor results in the practice [69]. To improve the performance of prediction of both continuous and discontinuous epitopes, machine learning methods were evolved. Most of these methods were developed based on very small datasets and used randomly selected peptides instead of experimentally verified nonepitopes as a negative training set [70]. Currently used methods for continuous epitope prediction combine two or more residue properties with machine learning approaches (summarized in Tables 2 and 3). In general, prediction methods can be divided based on the level of input information to methods based on antigen sequence and methods based on 3D structure of antigen. Structure-based methods significantly outperform sequence-based methods [71]. Unfortunately, existing prediction methods are not accurate enough and annotate general immunogenic/epitope-like regions on the antigen [69, 72]. It was demonstrated that consensus of various B-cell epitope prediction methods ensures greater accuracy of the results [73]. Here we offer a short overview of publicly available methods and servers for prediction of continuous as well as discontinuous B-cell epitopes (summarized in Tables 2 and 3).

### 4.1. Prediction of Continuous B-Cell Epitopes

The first prediction method using recurrent neural network, ABCPred, has been trained on B-cell epitopes obtained from BciPep database and nonepitopes obtained randomly from Swiss-Prot database. The ABCPred is a neural network based method for prediction of continuous B-cell epitopes using fixed length pattern [20]. The ABCPred dataset contains data of epitopes from viruses, bacteria, parasites, and fungi that are stored in BciPep database with the prediction accuracy of 65.9%. ABCPred, AAP method and BCPred, and BayesB predict only short peptide fragments. The B-cell epitopes of the Emy162 protein of* Echinococcus multilocularis* (the causative agent of zoonotic helminthosis) were predicted using BCPred and ABCPred [74].

APCPred combines amino acid anchoring pair composition (APC) and support vector machine (SVM) methods, which significantly improved the prediction accuracy. APCPred achieved an improved area under curve (AUC) of 0.794 [75]. BCPred server allows choosing prediction method among amino acids pair scaling method (AAP), BCPred, and FBCPred. AAP approach is based on the finding that particular amino acid pairs occur more frequently in epitope than nonepitope sequence. Combination of AAP propensity scale with turns, accessibility, antigenicity, hydrophilicity, and flexibility propensity scales improved the accuracy (72.5%).

BCPred method employs subsequence kernel-based SVM classifier and was trained on homology-reduced dataset of linear B-cell epitopes (with <80% sequence identity) derived from dataset previously used to test ABCPred. The performance of BCPred (AUC 0.758) outperforms implementation of AAP (AUC 0.7).

FBCPred is a novel method developed for prediction of B-cell epitopes with flexible length. Homology-reduced dataset is publicly available for comparing existing linear B-cell epitope prediction methods and testing of new prediction software.

BepiPred predicts continuous epitopes by combining two residues properties with Hidden Markov Model. BepiPred was evaluated on dataset of epitopes extracted from the literature, AntiJen, and HIV databases. This method has a quite low sensitivity [19].

The server BcePred is used for prediction of continuous B-cell epitopes based on physicochemical properties and allows user to select any residue property or combination of two or more properties employed in prediction. The performance of BcePred was evaluated on dataset containing epitopes obtained from BciPep database and dataset of randomly chosen nonepitopes from Swiss-Prot. The accuracy of BcePred combining four amino acid properties (hydrophilicity, flexibility, polarity, and exposed surface) is 58.70% [76].

A novel continuous B-cell epitope prediction method EPMRL was developed using multiple linear regression. EPMLR was tested on BEOD dataset containing only experimentally verified epitopes and nonepitopes and achieves overall sensitivity of 81.8% and precision of 64.1% and area under the receiver operating characteristic curve (AUC) of 0.728 [77].

B-cell epitope prediction using support vector machine tool (BEST) is sequence-based tool designed for prediction of both linear and conformational epitopes from full antigen sequence. Prediction is based on averaging of selected scores (sequence conservation, similarity to experimentally validated B-cell epitopes, predicted secondary structure, and relative solvent accessibility) generated from 20-mers. BEST achieves AUC at 0.81 and 0.85 for the fragment-based prediction and 0.57 and 0.6 for full antigen. BEST outperforms several modern sequence-based B-cell epitope predictors including ABCPred, BCPred, COBEpro, and CBTOPE [16].

SVMTriP employs support vector machine to combine the tripeptide similarity and propensity scores to predict linear epitopes. SVMTriP achieves a sensitivity of 80.1% and a precision of 55.2% and the AUC value 0.702, when tested on nonredundant epitopes extracted from IEDB [78]. A comparative study concluded that the methods based on sequence analysis do not predict epitopes better than chance. Since the majority of epitopes are discontinuous, prediction methods taking into account structural data could increase the accuracy of epitope prediction [69].

### 4.2. Prediction of Discontinuous B-Cell Epitopes

Although the majority (~90%) of the B-cell epitopes are discontinuous (conformational), to date much effort was concentrated on identification of continuous epitopes [22]. However, with the advance of proteomics and increasing number of Ag-Ab crystal structures available in databases, it is now easier to perform deeper analyses of conformational epitopes. These epitopes comprise linear stretches of residues brought into close proximity upon protein folding and the reconfiguration of epitope residues when an antigen is in complex with specific antibody. Most of the prediction methods are antibody-ignored methods. One must also take into account the fact that predicted epitopes are frequently short sequences of residues that represent the part of discontinuous epitope. The tools currently used for prediction of discontinuous epitopes are summarized in Table 3.

The first attempts at epitope prediction based on 3D structure began with development of CEP server, which is based on accessibility of amino acid residues and requires the 3D data in PDB format [17]. Hitherto, this tool is deprecated and is not available. Subsequent server, DiscoTope, predicts discontinuous B-cell epitopes by combining the surface accessibility and spatial and amino acid statistics to differentiate between epitopes and nonepitope sites. It generates one residue propensity score in the sphere of 10 Å which is the result of combination of the hydrophilicity scale and the epitope log-odds ratios [22]. DiscoTope has been recently updated to 2.0 version by Kringelum and coworkers with several improvements for proper benchmark definitions and use and achieves an AUC of 0.731 [71].

BEpro server (formerly known as PEPITO) uses a combination of amino acid propensity scores along with side chain orientation and solvent accessibility information using half sphere exposure values at multiple distances to predict discontinuous B-cell epitopes. It achieves AUC of 75.4 on the DiscoTope dataset [79]. BEpro and CEP prediction is based on the detection of exposed residues ignoring the residues buried in the spatial structure, which may affect the reliability of predictions.

PEPOP is structure-based method, which identifies clusters of accessible surface residues and segments that might form putative discontinuous epitopes and can be used to design immunogenic peptides. The anti-peptides antibodies showed reactivity with the cognate antigens in 80% of the cases (four cases from five) and were used in sandwich capture assay. Compared to CEP and DiscoTope, PEPOP showed comparable specificity and slightly better sensitivity [80]. Hitherto, this tool is deprecated and is not available.

Improved Spatial Epitope Prediction of Protein Antigens server (SEPPA) focusing on single residue propensity scales and continual segment clustering was developed in 2009 by Sun and colleagues [81]. SEPPA employs a novel concept of unit patch of residue triangle and spatial clustering coefficient to describe local spatial context in protein antigen surface and 3D characteristic of epitopes. A parameter of 4 Å was chosen in the definition of unit patch of residue triangle. Curated data of nonredundant spatial epitopes from PDB database was used for method testing. SEPPA outperforms popular prediction tools, CEP, DiscoTope, and BEpro, and achieves an average AUC over 0.742 [81].

Server ElliPro (derived from Ellipsoid and Protrusion) implements modified method for identifying continuous epitopes in the protein regions protruding from the globular surface of antigen [82] in combination with a residue clustering algorithm for prediction of discontinuous epitopes from primary antigen sequence or structure [83]. ElliPro performs BLAST search of PDB for antigen sequences homologues or use MODELLER [84] to predict 3D structure. ElliPro (AUC value of 0.732) outperforms structure-based methods CEP and DiscoTope. ElliPro enables visualization of linear and discontinuous epitopes on the protein 3D structure [83].

Computational prediction tool EPITOPIA employs Naïve Bayes classifier to predict epitopes in linear sequence or 3D structure. It distinguishes the nonepitope and epitope regions by computing an immunogenicity score (reflecting the immunogenic potential of a certain residue relative to all residues in the antigen) for each solvent accessible residue or a score for every amino acid. EPITOPIA yields higher success rate of 89.4% (mean AUC value of 0.60) when compared to ElliPro and DiscoTope [18].

CBTOPE was proposed for the prediction of discontinuous epitopes from antigen primary structure. This SVM-based predictor combines traditional features of physicochemical profiles and sequence-derived inputs including composition and collocation of amino acids. It outperformed other structure methods using binary profile of pattern and physicochemical profile of patterns with better sensitivity and AUC on the same benchmark dataset [85].

Epitope prediction method, which uses Consensus Scoring (EPCES) combines scores from residue epitope propensity, residue conservation, side-chain energy, contact number, surface planarity, and secondary structure composition. EPCES predicts discontinuous epitopes with 47.8% sensitivity, 69.5% specificity, and an AUC value of 0.632, which is statistically similar to other published methods.

The Antigenic Epitopes Prediction with Support Vector Regression server (EPSVR) employs vector regression to integrate same scores as are combined in EPCES and achieves AUC value of 0.597. EPSVR is integrated in metaserver EPMeta together with five existing prediction servers (EPCES, EPITOPIA, SEPPA, PEPITO, and DiscoTope 1.2) and provides consensus prediction results. The performance of EPMeta is AUC value of 0.6, which is higher than performance of any other existing single server. Unfortunately, this server met unsolvable technical difficulties and is no more available.

Evaluation of performance of prediction tools is often difficult, especially when each of them has their own testing dataset. To solve this problem and help users to choose the tool, the recent web servers, CEP [17], DiscoTope [22], PEPOP [80], ElliPro [83], BEpro [79], and SEPPA [81], were tested with an independent dataset created by collection of the experimentally confirmed discontinuous epitopes. SEPPA gave the best performance among the six tools (the averaged AUC value of 0.62, sensitivity of 0.49) followed by DiscoTope and BEpro (the averaged AUC value of 0.58 and 0.55, sensitivity of 0.36 and 0.18). The performance of CEP, PEPOP, and ElliPro did not exceed averaged AUC values of 0.55 [86]. The detection based on exposed residues ignoring the residues buried in the structure can account for low performance of CEP tool. The best performance achieved by SEPPA could be attributed to growing number of available structural data and new spatial features incorporated in its algorithm [86].

### 4.3. Antibody-Specific Epitope Prediction

The traditional antibody-ignored epitope prediction methods do not take into account the reconfiguration of epitope residues when an antigen is in complex with a specific antibody [3]. Reconfiguration of Ag takes place when Ab binds both short peptide or whole antigen. To reflect this biological reality, several prediction methods based on sequence or structure of interacting Ab and Ag have been introduced in the last few years. The performance of antibody-based prediction of epitopes is competitive, or even better, when compared with structure-based predictors (rigid-body docking algorithms) [3].

A method using Antibody-Specific Epitope Prediction (ASEP) index developed by Soga and coworkers, represents the first benchmark in epitope prediction for individual antibody and has been used to narrow down candidate epitopes previously predicted by the conventional methods [87].

The EpiPred combines conformational matching of the Ab-Ag structures and knowledge based asymmetric Ab-Ag scoring to annotate the likely epitope regions specific to the given antibody [72]. This global docking pipeline requires the sequence of Ab and structure of unbound Ag. Compared to rigid-body docking algorithms, EpiPred significantly enriches the number of close-to-native decoys when adjusting the Ab sequence against the Ag [72].

Predicting Epitopes Using Antibody Sequences (PEASE) evaluates a pair score for all combinations of one residue from the complementarity determining regions (CDR) of antibody and one residue from the surface exposed region of antigen. A residue score of antigen surface residue is its highest pair score. A higher residue score means that contact between antibody and antigen residue is more strongly predicted and that this residue constitutes a part of B-cell epitope. PEASE also identifies surface patches on the antigen, which contain multiple residues with high residue scores [88]. PEASE was successfully used to predict the vaccinia virus epitopes [89].

B-cell epitope prediction through association rules (Bepar) is predicting epitopes based on antibody-antigen (paratope-epitope) association patterns which can be applied to any antibody-antigen sequence pair. Residue cooperativity and relative composition have been used to enhance the performance of this method. Bepar shows competitive performance on epitope prediction and outperforms CEP even without antigen 3D structure information [90].

### 4.4. Mimotope-Based Epitope Prediction

In recent years, the epitope prediction methods employing mimotopes derived from phage display experiments were developed. In general, these methods can be classified as methods that map mimotopes to the overlapping location patches on the antigen surface using statistical features of mimotopes and methods using mimotope mapping back to the antigen sequence through alignment. Mimotope has similar physicochemical properties and spatial organization but however rarely shows sequence similarity to the native antigen. In some cases, mimotope mapping back to the antigen can indicate B-cell epitope location [91]. B-cell epitope prediction tools based on mimotope analysis are summarized in Table 4.

## 5. Conclusion

Antibodies are currently the most promising class of biopharmaceuticals. The main objective of epitope identification is to replace an antigen in the immunization, antibody production, and serodiagnosis. The accurate identification of B-cell epitopes and large-scale data integration still presents major challenges for immunologists. Advances in B-cell epitope mapping and computational prediction have yielded molecular insights into the process of biorecognition and formation of Ag-Ab complex, which may help to formulate even more precise algorithms to predict their localization in the antigen. However, based on statistics it is not possible to precisely determine the epitope characteristics, which allow biorecognition. One has to keep in mind that the epitopes are not intrinsic feature of proteins and antibody-ignored prediction methods predict only putative epitope to which an undefined Ab might bind. The real epitopes cannot be predicted ignoring the structural effect upon Ag-Ab complex formation. This fact opens new space for all algorithms to improve further.

## Figures and Tables

**Table 1:** List of B-cell epitope databases.

Database	Source (URL)	Ref.
IEDB IEDB-3D	http://www.iedb.org/	—
AntiJen	http://www.ddg-pharmfac.net/antijen/AntiJen/antijenhomepage.htm	[56, 57]
CED	http://immunet.cn/ced/	[59]
Epitome	http://www.rostlab.org/services/epitome/	[60]
BciPep	http://www.imtech.res.in/raghava/bcipep/info.html	[61]
SEDB	http://sedb.bicpu.edu.in	[62]
SDAP	https://fermi.utmb.edu/	[63]
HIV Molecular Immunology Database	http://www.hiv.lanl.gov/content/immunology/index.html	—
FLAVIdB	http://cvc.dfci.harvard.edu/flavi/	[64]

**Table 2:** List of web available tools for continuous B-cell epitope prediction.

Tool	Source (URL)	Input data	Ref.
ABCPred	http://www.imtech.res.in/raghava/abcpred/	FASTA	[20]
APCPred	http://ccb.bmi.ac.cn/APCpred/	FASTA	—
BCPREDS	http://ailab.ist.psu.edu/bcpred/	FASTA	[92, 93]
BepiPred	http://www.cbs.dtu.dk/services/BepiPred	FASTA or FASTA file	[19]
LBtope	http://crdd.osdd.net/raghava/lbtope/	FASTA or FASTA file	[94]
Bcepred	http://www.imtech.res.in/raghava/bcepred/	FASTA or FASTA file	[76]
SVMTriP	http://sysbio.unl.edu/SVMTriP/	FASTA	[78]

**Table 3:** List of web available tools for discontinuous/conformational B-cell epitope prediction.

Tool	Source (URL)	Input data	Ref.
DiscoTope	http://www.cbs.dtu.dk/services/DiscoTope-2.0/	PDB ID or PDB file	[22, 71]
BePro (PEPITO)	http://pepito.proteomics.ics.uci.edu/	PDB ID or PDB file	[79]
ElliPro	http://tools.immuneepitope.org/ellipro/	FASTA or Swiss-Prot ID	[83]
SEPPA	http://badd.tongji.edu.cn/seppa/	PDB ID or PDB file	[81]
EPITOPIA	http://epitopia.tau.ac.il/	FASTA/PDB ID or PDB file	[95]
CBTOPE	http://www.imtech.res.in/raghava/cbtope/	FASTA or FASTA file	[85]
EPCES	http://sysbio.unl.edu/EPCES/	PDB ID or PDB file	[96]
EPSVR	http://sysbio.unl.edu/EPSVR/	PDB ID or PDB file	[97]
PEASE	http://www.ofranlab.org/PEASE	Ag PDB ID or PDB file Ab FASTA or FASTA file	[88]
EpiPred	http://opig.stats.ox.ac.uk/webapps/sabdab-sabpred/EpiPred.php	PDB ID or PDB file	[72]

**Table 4:** List of B-cell epitope prediction tools based on mimotope analysis.

Tool	Source	Ref.
MIMOX	http://immunet.cn/mimox/	[91]
MimoPro	http://informatics.nenu.edu.cn/MimoPro http://informatics.nenu.edu.cn/PepMapper/	[98]
Pep-3D-Search	http://informatics.nenu.edu.cn/PepMapper/	[99]
MIMOP	upon request	[13]
LocaPep	http://atenea.montes.upm.es/#soft	[100]
PepSurf	http://pepitope.tau.ac.il/sources.html	[101]
